# Authentication of *Puerariae Lobatae Radix* and Its Adulterant *Puerariae Lobatae Caulis* via Digital ID Technology

**DOI:** 10.3390/foods15132344

**Published:** 2026-07-02

**Authors:** Tianxin Ma, Zhixin Jia, Xianrui Wang, Haonan Wu, Yongqiang Lin, Huijun Li, Jia Chen, Xianlong Cheng

**Affiliations:** 1 Institute for Control of Traditional Chinese Medicine and Ethnic Medicine, National Institutes for Food and Drug Control, Beijing 102629, China; 16637762696@163.com (T.M.); jiazhixin@nifdc.org.cn (Z.J.); xianruiwang@nifdc.org.cn (X.W.); 19861403424@163.com (H.W.); linyongqiang@nifdc.org.cn (Y.L.); 2 School of Traditional Chinese Pharmacy, China Pharmaceutical University, Nanjing 211198, China; cpuli@163.com; 3 State Key Laboratory of Drug Regulatory Science, Beijing 102629, China

**Keywords:** *Puerariae Lobatae Radix*, *Puerariae Lobatae Caulis*, UPLC-QE-Orbitrap-MS, digital ID

## Abstract

*Puerariae Lobatae Radix* (PLR) is a commonly used herbal medicine in traditional Chinese medicine, whereas *Puerariae Lobatae Caulis* (PLC), its aerial part, is frequently utilized as an adulterant due to similar morphological features. Conventional identification methods and pharmacopoeial criteria fail to effectively distinguish these two homologous herbs, creating a demand for a precise identification method. Therefore, a Digital ID method was established for their identification. UPLC-QE-Orbitrap-MS coupled with Progenesis QI software was applied to analyze multi-batch samples of PLR and PLC. Common ion information was extracted, and a differential ion matrix was constructed after eliminating blank ions and cross-shared components. High-intensity characteristic ions were defined as exclusive Digital ID for authenticity identification. The Matching Confidence (MC) values of both herbs matching their own Digital IDs were >85%, while cross-matching MC values were <4%. Unlike subjective macroscopic authentication, compendial TLC and single-marker quantification that fail to discriminate the two materials, and spectroscopic methods incompatible with powdered samples, this digital ID framework enables automated objective authentication via quantitative Matching Confidence without manual spectral comparison. This technique achieves accurate and efficient digital identification of the two herbs, facilitating the quality control of PLR and offering a new strategy for the quality assessment of traditional Chinese medicine (TCM).

## 1. Introduction

*Puerariae Lobatae Radix* is the dried root of *Pueraria lobata* (Willd.) Ohwi (Leguminosae). It was first documented in Shennong’s Classic of Materia Medica, and possesses the efficacies of relieving muscles to abate fever and promoting fluid production to quench thirst. Clinically, it is commonly used for the treatment of exogenous fever with headache, stiffness and pain in the nape and back, consumptive thirst, incomplete measles eruption, febrile dysentery, etc. [1,2]. Modern pharmacological studies have demonstrated that its active constituents can regulate the TAK1-mediated TLR4/NF-κB signaling pathway, exerting effects in alleviating rheumatoid arthritis and preventing osteoporosis [3,4]. PLC refers to the aerial stems of Pueraria lobata. As recorded in the Compendium of Materia Medica, PLC is mainly indicated for acute laryngeal impediment, which is drastically different from the efficacies of PLR (relieving muscles to abate fever, promoting fluid production to quench thirst, and promoting eruption). Furthermore, it is not officially collected in the Chinese Pharmacopoeia. Owing to its homologous origin with PLR, PLC, which is easily available and of low cost, is frequently misidentified or deliberately adulterated into PLR. Although these two herbs can be distinguished by morphological characteristics, their diagnostic features become obscure after being cut into small pieces, posing difficulties in accurate identification for non-professionals [5,6]. PLC also contains high levels of puerarin, whose content exceeds the limit specified in the Chinese Pharmacopoeia [7,8], rendering the current quality standards incapable of effectively identifying such adulteration. The application of PLR adulterated with PLC not only fails to achieve the expected therapeutic efficacy but also compromises medication safety, and further hinders the standardized and healthy development of the Chinese materia medica industry [9]. Therefore, it is urgent to establish rapid and efficient methods for the identification and analysis of these two herbs.

Traditional identification methods for PLR mainly include morphological identification, microscopic identification, physicochemical identification, and other approaches. However, the thin-layer chromatography (TLC) identification and content assay stipulated under the item of PLR in the ChP 2025 Edition fail to distinguish between these two medicinal materials [1]. With the emergence of various new technologies and methodologies, diverse technical means have been applied to the identification of PLR and its adulterant PLC. For example, Zhang et al. [10] adopted hyperspectral imaging technology combined with multi-layer perceptron (MLP), partial least squares discriminant analysis (PLS-DA) and support vector machine (SVM) models, which could effectively differentiate PLR from its counterfeit PLC with an accuracy of up to 97.74%. Qiu et al. [11] utilized near-infrared spectroscopy integrated with machine learning algorithms to discriminate and quantify adulterated PLR, achieving a 100% discrimination rate. Although these methods enable the identification of PLR and PLC to a certain extent, they also have several limitations. Morphological identification mainly relies on the experience and sensory judgment of identifiers, which is highly subjective [12]. Hyperspectral technology can realize non-destructive and rapid identification of herbal decoction pieces, but it cannot be used for the identification of crude drug powders or compound preparations. Liquid chromatography–mass spectrometry (LC-MS) technology allows the detection of multiple components, and its combination with chemometrics enables the analysis of the chemical constituents contained therein. As an emerging innovative concept in the field of digital identification of TCM, the “Digital ID” relies on high-resolution mass spectrometry (HRMS) technology: mass spectrometric analysis is first performed on multiple batches of the same medicinal herb to extract common component ions and establish a quantitative matrix; then the common ionic components of different medicinal herbs are removed, and characteristic ions with top-ranked ion intensities are selected to form a specific identification marker; finally, quantitative analysis of the matching degree between test samples and the digital marker is conducted on the basis of MC [13,14].

In this study, PLR and PLC were distinguished based on UHPLC-Q-Exactive Orbitrap MS combined with the “Digital ID” strategy. A “Characteristic Ion Set” of PLRand PLC was established, which refers to an ordered matrix of mass spectrometric data for differentiating the two medicinal materials. Furthermore, this “Characteristic Ion Set” was applied to achieve accurate identification of unknown samples. The proposed technical method can realize the effective discrimination and identification of PLR and PLC, providing scientific data support for ensuring the medicinal safety of PLC, and offering a referential research paradigm for the studies on adulteration identification of other Chinese materia medica.

## 2. Materials and Methods

### 2.1. Samples

A total of 20 batches of PLR and PLC samples were obtained from the National Institutes for Food and Drug Control (NIFDC), which were collected from different production areas including Hubei, Henan, and Hunan provinces, as detailed in Table 1. All samples were authenticated by Researcher Xianlong Cheng from the Institute of Chinese Materia Medica as genuine PLR and PLC, respectively. Among these samples, 16 batches (8 batches of PLR and 8 batches of PLC) were selected for the establishment of the “Digital ID” of the two herbs, and the remaining 4 batches were used for the final identification and matching verification.

### 2.2. Reagents

Ultra-pure water was produced by a Milli-Q water purification system. HPLC-grade acetonitrile (Thermo Fisher Scientific, purity > 99.9%, Beijing, China), HPLC-grade formic acid (Dikma Technologies, purity > 99%, Beijing, China), and HPLC-grade methanol (Thermo Fisher Scientific, Beijing, China) were used in this study.

### 2.3. Instruments

Thermo Q Exactive high-resolution liquid chromatography–tandem mass spectrometry system (Thermo Fisher Scientific, Waltham, MA, USA); KQ-500DE ultrasonic cleaner (Kunshan Ultrasonic Instrument Co., Ltd., Kunshan, China); and XS105DU and XS205DU electronic analytical balances (readability: 0.01 mg, METTLER TOLEDO, Zurich, Switzerland).

## 3. Methods

### 3.1. Sample Pretreatment

The above samples were ground into fine powder using a grinder and sieved through a No. 3 sieve (Particle size: 355 ± 13 μm). An accurately weighed 1 g portion of the powder was placed into a 100 mL conical flask with a stopper, followed by the addition of 50 mL of 50% methanol. The mixture was shaken thoroughly and weighed, then subjected to ultrasonic extraction (500 W, 40 kHz) for 30 min. After cooling to room temperature, the weight loss was replenished with 50% methanol. The solution was filtered, and the subsequent filtrate was collected for analysis.

### 3.2. UHPLC-Q-Exactive Orbitrap MS Analysis

Chromatographic conditions: The separation was conducted on a Waters ACQUITY UPLC HSS T3 column (100 mm × 2.1 mm, 1.8 μm) with an injection volume of 2 μL. The mobile phase consisted of acetonitrile (A) and 0.1% formic acid in water (B) at a flow rate of 0.3 mL/min. The gradient elution program was set as follows: 0–5 min, 95% B; 5–10 min, 95–92% B; 10–15 min, 92–88% B; 15–20 min, 88–85% B; 20–25 min, 85–75% B; 25–30 min, 75–60% B; 30–32 min, 60–20% B; 32–36 min, 20% B; 36–36.1 min, 20–95% B; and held at 95% B until 40 min.

Mass spectrometric conditions: Primary high-resolution mass data and secondary mass spectra were acquired in both positive and negative electrospray ionization (ESI) modes. The operating parameters were set as follows: spray voltage of 3.5 kV, capillary temperature of 350 °C, sheath gas flow rate of 35 arb, and auxiliary gas flow rate of 10 arb. The mass scanning range was *m*/*z* 150–1500 with a mass resolution of 70,000. The secondary mass data were collected in the dd-MS2/dd-SIM mode at a resolution of 17,500, and the collision voltage ranged from 10 to 50 V.

### 3.3. Data Processing

The mass spectrometry data files of PLR, PLC and 50% methanol (blank solvent) were quantitatively processed using Progenesis QI 2.3 software. A data matrix comprising retention time (*t*), mass-to-charge ratio (*m/z*) and ion abundance (i) was generated and saved in .csv format. Further data processing was performed using Java scripts.

(1) Blank Solvent Ion Elimination

The mass spectral data of test samples may contain background signals derived from the blank solvent, which would interfere with the experimental results. Therefore, elimination of these interfering ions is required to enhance the accuracy of the analysis. Ions in the test samples were regarded as homologous to those in the blank solution and were removed if the retention time difference (Δ*t*) ≤ 0.10 min and the mass-to-charge ratio difference (Δ*m*/*z*) ≤ 0.01 Da.

(2) Acquisition of Common Ion Matrix

Variations in chemical compositions may exist among different batches of the same medicinal herb, leading to discrepancies in mass spectral data. The mass spectral data of all batches for the same herb were imported and aligned. Ions with a retention time difference (Δ*t*) ≤ 0.10 min and a mass-to-charge ratio difference (Δ*m*/*z*) ≤ 0.01 Da were defined as identical ions and stored as common ions. All common ions were integrated to construct a common ion matrix. The common ion matrix of PLR was named PLO, and that of PLC was designated as PML.

(3) Acquisition of Differential Ion Matrix

Both PLR and PLC originate from *Pueraria lobata* (Willd.) Ohwi or *Pueraria thomsonii* Benth. (Leguminosae), representing different medicinal parts of the same plant, thus sharing similarities in their chemical constituents. To obtain differential ions, the intersecting ions between PLR and PLC were removed. Specifically, homologous ions in PLO and PML were excluded from their respective matrices, generating individual differential ion matrices for each herb.

(4) Establishment of the “Digital ID”

From the differential ion matrices obtained in step (3), the top 50 ions ranked by ion intensity were separately selected to construct new ion matrices for each herb, namely the “Digital ID”.

(5) Identification Using Digital Method

The “Digital ID” profiles of PLO and PML were used to match the blind samples, and the MC was calculated. The formula for MC is defined as follows:

$$\mathrm{MC}\;=\;\frac{Number\;of\;successfully\;matched\;ions}{Total\;number\;of\;ions\;in\;the\;“Digital\;ID”}\times 100\%$$

## 4. Experiment Results

### 4.1. UPLC-QE-MS Analysis

**Figure 1:**
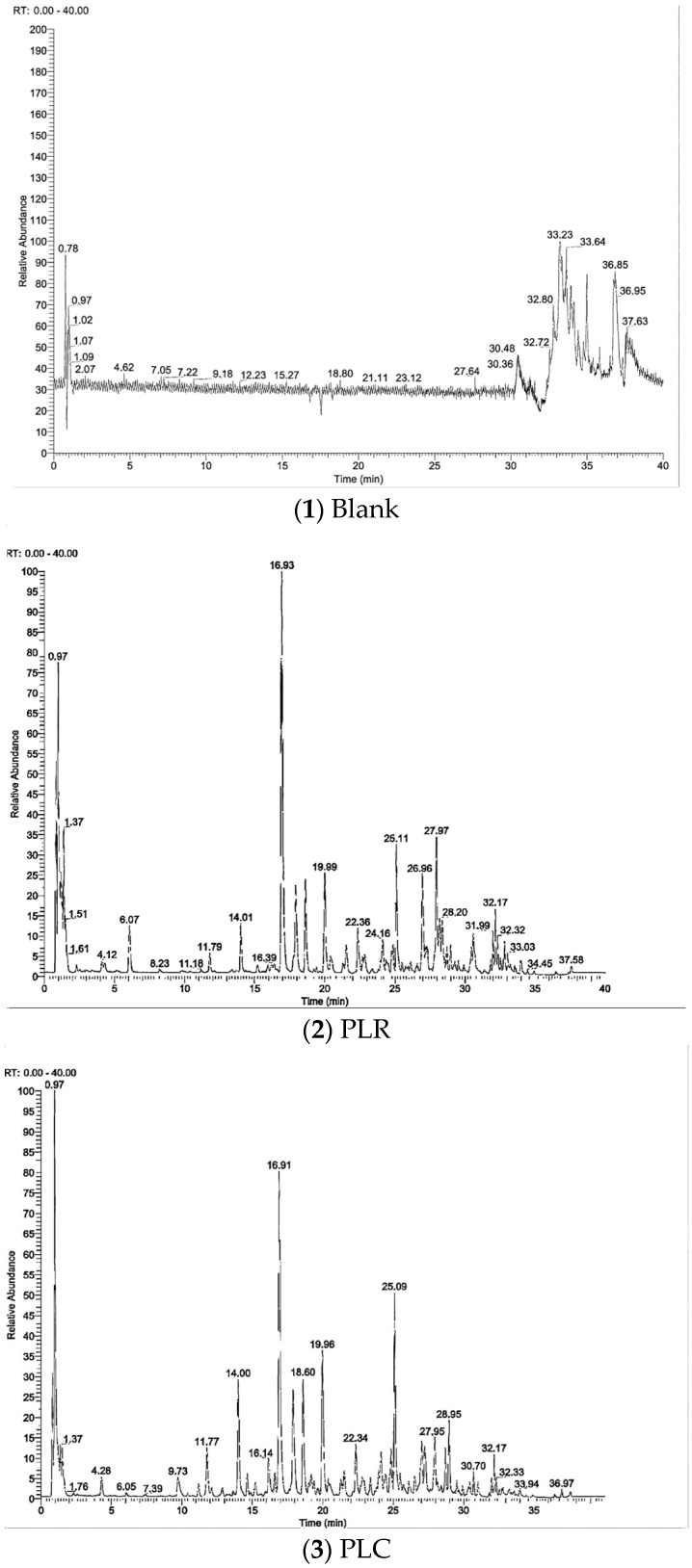
The mass spectrograms of blank, PLR and PLC.

Based on the aforementioned sample preparation, chromatographic and mass spectrometric methods, mass spectral profiles (total ion chromatograms, TICs) of the blank solvent, PLR and PLC were acquired, as depicted in Figure 1. As PLR and PLC originate from the same plant, they show negligible differences in their chemical compositions. While the contents of their common components vary, the fold differences are relatively small. Therefore, they cannot be effectively distinguished from the TICs alone, and subsequent digital analysis is required.

### 4.2. Digital Quantitative Processing

Based on the data processing method detailed in Section 2.3, after blank subtraction, the quantities of [tR-*m*/*z*-I] features for the eight batches of samples utilized to extract the “Digital IDs” of PLR and PLC are presented in Table 2. As shown in Table 2, the eight distinct batches of PLR and PLC possess varying numbers of [tR-*m*/*z*-I] features, which indicates the differences in the chemical compositions of different batches. These discrepancies in chemical constituents are most likely attributed to the fact that they are derived from different parts of the same plant.

### 4.3. Digital Identification of PLR and PLC

The mass spectrometry data of PLR and PLC were digitized using Progenesis QI software, and the information of the top 50 ions ranked by ion intensity was extracted as their respective “Digital IDs”. The retention times [tR-*m*/*z*-I], mass-to-charge ratios (*m*/*z*), and ion intensities of the top 50 specific ions in the “Digital IDs” of PLR and PLC are presented in Table 3 and Table 4, respectively.

**Figure 2:**
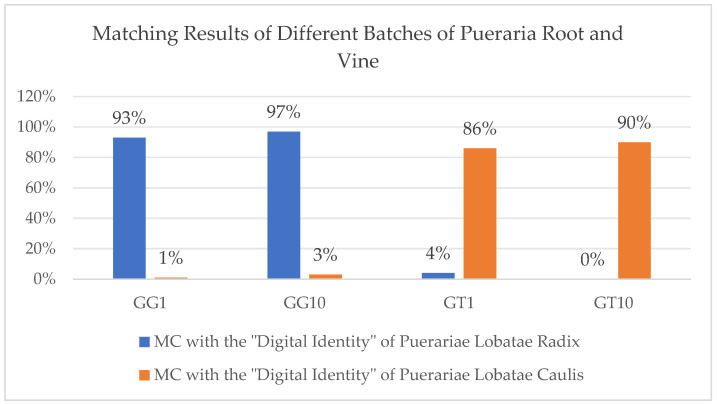
Matching and identification results of different batches of PLR and PLC.

The mass spectral data matrix of the test samples was matched with the reference “Digital IDs” of PLR and PLC, and MC was evaluated accordingly. The identification results of two batches of PLR and two batches of PLC are displayed in Figure 2. The Matching Confidence values between the test samples of PLR and its own “Digital ID” were all > 92%, while those between the test samples of PLC and its own “Digital ID” were all > 85%. Sample GG10 achieved the highest MC value of 97%. Conversely, the matching degrees between the test samples of PLR and the “Digital ID” of PLC were all < 4%, among which batch GG1 showed the lowest MC value of 1%. Similarly, the Matching Confidence values between PLC and the “Digital ID” of PLR were <4%, and the test sample batch GT10 exhibited an MC value of 0%. It is evident that there are significant differences in the Matching Confidence between PLR and PLC, demonstrating the feasibility of distinguishing these two herbs using their “Digital IDs”.

Based on the matching results between PLR, PLC and their respective “Digital IDs”, significant differences in Matching Confidence were observed, enabling the effective identification and differentiation of these two medicinal materials. This demonstrates that the “Digital IDs” of PLR and PLC exhibit favorable specificity. By utilizing these established “Digital IDs”, the digital identification of PLR and PLC can be accomplished accurately and efficiently.

## 5. Discussion

The authentication of TCM is a critical procedure for guaranteeing the safety and efficacy of clinical medication, and also serves as the cornerstone for the internationalization and modernization of TCM. The problems of therapeutic and safety risks caused by the adulteration of different medicinal parts derived from the same plant are representative, and how to identify such adulteration is one of the difficulties in the quality control of TCM [15,16]. PLR and PLC are hard to distinguish under the current quality standards due to their similar botanical origins, morphological traits and chemical constituents. Traditional identification methods fail to achieve accurate discrimination, which brings potential risks to clinical medication. In this study, a “Digital ID” system was constructed based on UPLC-QE-Orbitrap-MS technology to realize the digital identification of these two herbs, providing a new strategy for the authentication of TCM.

The optimization of sample pretreatment and detection conditions is the foundation for ensuring the accuracy of identification. In this research, the extraction effects of solvents including methanol, 40% methanol, 50% methanol and 70% methanol were comprehensively investigated, and 50% methanol was ultimately determined as the optimal extraction solvent. During the development of the mass spectrometric method, by comparing the peak profiles and ion counts in positive and negative ion modes, it was found that the negative ion mode presented better spectral quality and more comprehensive ion detection, providing high-quality data support for the subsequent screening of differential ions. The mass spectrometry data were quantitatively processed using Progenesis QI software. Through a series of procedures including blank ion elimination, common ion screening and differential ion extraction, the top 50 specific ions ranked by ion intensity were finally selected to construct the “Digital ID”. In the blank ion elimination step, the screening thresholds of Δt ≤ 0.10 min and Δ*m*/*z* ≤ 0.01 Da were set to effectively exclude solvent interference; the common ion screening avoided the influence of constituent fluctuations in herbal materials of different batches caused by producing areas and growth environments. We selected puerarin with *m*/*z* of 415.1024 as the characteristic marker compound of the Pueraria lobata sample, and injected the identical sample solution six times continuously under consistent instrumental and chromatographic conditions to assess the reproducibility of its peak area response. Statistical calculation showed that the relative standard deviation (RSD) of the puerarin peak area was only 1.6% across six replicate injections. Such a low RSD value fully indicates that the reproducibility of the whole LC-MS detection workflow proposed in this manuscript is satisfactory and completely meets the standard requirements for stable sample measurement. The identification results showed that the MC values of the test samples of PLR and PLC against their own “Digital IDs” were all > 85%, while the MC values against the “Digital ID” of the counterpart were all < 5%, and some were even 0%, which fully proved that the “Digital ID” has strong specificity and can realize the accurate differentiation of the two herbs.

Compared with existing identification technologies, the method in this study has significant advantages. Traditional morphological identification relies on empirical judgment, which is highly subjective and easily affected by the processing state of samples; although TLC identification and content determination are pharmacopoeial methods, they cannot distinguish counterfeits with highly similar components such as PLR and PLC. Technologies such as hyperspectral imaging can achieve rapid identification, but they cannot intuitively characterize differences in chemical components and are inapplicable to powdered samples, lacking application extensibility. In contrast, the combination of HRMS with the “Digital ID” technology not only exerts the advantages of high-resolution LC-MS in multi-component, high-sensitivity and high-accuracy detection, but also realizes the digitization and objectification of identification standards through the construction of a characteristic ion matrix, avoiding human errors, and the operational process can be standardized and popularized [17].

This study still has certain limitations: for example, the samples only cover three producing areas (Hubei, Henan and Hunan provinces), and the scope of producing areas and sample batches needs to be expanded in the follow-up to further verify the universality of the “Digital ID”; the screening of the 50 characteristic ions in the “Digital ID” was based on ion intensity without fully considering the correlation with the pharmacological activities of the components, and the combination of characteristic ions can be optimized through combination with pharmacodynamic studies in the future.

## 6. Conclusions

In conclusion, the combination of HRMS and the “Digital ID” technology realizes accurate and efficient discrimination between PLR and PLC in this study. This established strategy can serve as a reliable technical solution for routine quality control of PLR. As a preliminary exploratory framework, it may provide reference ideas for the identification of other TCM species featuring highly similar chemical profiles and difficult discrimination, though further systematic verification across more medicinal varieties is still required to confirm its universal applicability. The proposed workflow lays a foundation for advancing the digitalization and standardization of TCM quality evaluation.

## Figures and Tables

**Table 1:** Sample information of PLR and PLC.

Sample	Batch No.	Origin	Purpose
*Puerariae Lobatae Radix* (PLR)	GG1	Hong’an City, Hubei Province	Identification and Analysis
	GG2	Xin County, Henan Province	Database Establishment
	GG3	Hong’an City, Hubei Province	Database Establishment
	GG4	Hong’an City, Hubei Province	Database Establishment
	GG5	Jinlong Village, Jishou, Xiangxi, Hunan Province	Database Establishment
	GG6	Jinlong Village, Jishou, Xiangxi, Hunan Province	Database Establishment
	GG7	Jinlong Village, Jishou, Xiangxi, Hunan Province	Database Establishment
	GG8	Jinlong Village, Jishou, Xiangxi, Hunan Province	Database Establishment
	GG9	Jinlong Village, Jishou, Xiangxi, Hunan Province	Database Establishment
	GG10	Jinlong Village, Jishou, Xiangxi, Hunan Province	Identification and Analysis
*Puerariae Lobatae Caulis* (PLC)	GT1	Hong’an City, Hubei Province	Identification and Analysis
	GT2	Xin County, Henan Province	Database Establishment
	GT3	Hong’an City, Hubei Province	Database Establishment
	GT4	Hong’an City, Hubei Province	Database Establishment
	GT5	Jinlong Village, Jishou, Xiangxi, Hunan Province	Database Establishment
	GT6	Jinlong Village, Jishou, Xiangxi, Hunan Province	Database Establishment
	GT7	Jinlong Village, Jishou, Xiangxi, Hunan Province	Database Establishment
	GT8	Jinlong Village, Jishou, Xiangxi, Hunan Province	Database Establishment
	GT9	Jinlong Village, Jishou, Xiangxi, Hunan Province	Database Establishment
	GT10	Jinlong Village, Jishou, Xiangxi, Hunan Province	Identification and Analysis

**Table 2:** Number of [tR-*m*/*z*-I] features in PLR and PLC.

Sample	Batch No.	Number
*Puerariae Lobatae Radix*	GG2	10,485
	GG3	10,396
	GG4	10,535
	GG5	10,516
	GG6	8199
	GG7	9184
	GG8	9486
	GG9	9535
*Puerariae Lobatae Caulis*	GT2	11,146
	GT3	10,604
	GT4	10,889
	GT5	10,309
	GT6	9470
	GT7	9109
	GT8	9421
	GT9	9517

**Table 3:** TOP 50 specific ion information of the “Digital ID” for PLR.

tR/min	*m*/*z*	I	tR/min	*m*/*z*	I
28.78	1029.49	32,863,014	32.02	997.50	2,901,496
17.64	207.07	29,188,807	25.97	283.06	2,851,187
16.09	207.07	24,924,998	22.88	829.20	2,642,240
16.81	711.16	23,072,122	29.25	979.48	2,556,123
17.72	295.05	21,845,075	30.57	283.06	2,264,817
27.70	987.48	20,194,249	25.54	829.20	2,254,026
27.70	987.48	19,043,693	28.50	913.98	2,248,875
17.71	295.05	17,266,159	23.29	569.11	2,208,684
29.61	957.50	14,692,390	15.18	751.17	2,102,463
29.61	957.50	13,748,104	4.11	847.23	2,080,138
27.56	1133.54	10,337,389	27.75	1103.53	2,064,048
27.55	1133.54	9,465,550	19.99	417.12	2,044,146
29.26	979.48	8,060,415	29.25	979.98	2,035,999
17.64	192.04	6,744,785	32.14	309.17	2,027,413
29.26	979.98	6,434,107	32.13	1265.67	2,016,691
19.93	637.14	6,076,358	30.31	1115.56	2,012,883
15.22	751.17	5,279,268	29.76	979.47	2,006,151
19.98	637.14	4,414,292	4.07	847.23	1,986,942
29.25	979.48	3,938,796	22.30	725.14	1,986,926
30.42	981.23	3,743,843	19.96	417.12	1,972,699
29.13	1009.47	3,591,600	25.53	829.20	1,847,074
4.08	847.23	3,237,378	23.26	569.11	1,843,379
29.25	979.98	3,159,125	25.68	525.12	1,776,582
29.26	899.46	3,022,291	29.27	971.99	1,775,332
30.84	1267.61	2,937,290	22.88	829.20	1,774,435

**Table 4:** TOP 50 specific ion information of the “Digital ID” for PLC.

tR/min	*m*/*z*	I	tR/min	*m*/*z*	I
31.37	969.43	21,401,667	20.01	697.20	2,741,831
27.99	957.47	18,184,787	31.37	969.43	2,599,576
27.99	957.47	17,914,215	34.68	419.19	2,588,451
16.51	549.15	16,118,896	20.03	697.20	2,539,541
31.37	969.43	10,287,659	34.68	419.19	2,539,131
16.51	549.15	9,453,454	29.73	957.51	2,536,681
14.29	627.19	5,565,803	30.90	1027.55	2,495,395
34.95	863.50	5,416,764	14.27	627.19	2,444,890
34.95	863.50	5,036,070	28.00	1073.53	2,442,306
14.29	690.19	4,696,476	20.01	697.20	2,425,271
28.09	957.47	4,407,540	28.38	1059.54	2,382,016
34.94	863.50	4,230,404	34.46	849.49	2,224,345
20.03	697.20	4,145,266	34.95	863.50	2,214,532
20.03	697.20	4,019,723	28.00	1073.53	2,203,792
18.02	745.26	3,883,548	26.34	353.03	2,184,142
14.68	725.19	3,512,947	18.05	745.25	2,168,629
20.02	697.20	3,490,796	34.94	863.50	2,167,021
18.04	745.25	3,094,708	34.10	471.35	2,138,688
27.98	957.47	3,036,547	14.65	725.19	2,071,947
16.50	549.15	2,996,081	11.57	627.19	1,984,889
34.70	419.19	2,863,451	34.11	471.35	1,918,831
20.03	697.20	2,855,087	23.24	613.21	1,906,311
27.99	957.47	2,827,740	34.55	471.35	1,897,084
34.11	471.35	2,792,214	26.37	353.03	1,871,980
14.30	627.19	2,779,159	28.00	1073.53	1,867,357

## Data Availability

The original contributions presented in this study are included in the article. Further inquiries can be directed to the corresponding authors.

## Abbreviations

This manuscript adopts the following abbreviations.

MC	matching confidence
TCM	traditional Chinese medicine
MLP	multi-layer perceptron
PLS-DA	partial least squares discriminant analysis
SVM	support vector machine
LC-MS	liquid chromatography–mass spectrometry
HRMS	high-resolution mass spectrometry
NIFDC	National Institutes for Food and Drug Control
